# Limited association between HRR gene alterations and HRD in molecular tumor board cancer samples: Who should be tested for HRD?

**DOI:** 10.1002/ijc.35457

**Published:** 2025-04-25

**Authors:** Christoph Schubart, Lars Tögel, Maria Giulia Carta, Philip Hetzner, Lina Helbig, Charlotte Zaglas, Maria Ziegler, Robert Stöhr, Annett Hölsken, Juliane Hoyer, Fulvia Ferrazzi, Clemens Neufert, Sebastian Lettmaier, Marianne Pavel, Henriette Golcher, Sarina K. Mueller, Florian Fuchs, Carla E. Schulmeyer, Matthias W. Beckmann, Bernd Wullich, Abbas Agaimy, Andre Reis, Arndt Hartmann, Norbert Meidenbauer, Silvia Spoerl, Florian Haller, Evgeny A. Moskalev

**Affiliations:** ^1^ Institute of Pathology Universitätsklinikum Erlangen, Friedrich‐Alexander‐Universität Erlangen‐Nürnberg (FAU) Erlangen Germany; ^2^ Comprehensive Cancer Center Erlangen‐EMN (CCC ER‐EMN) Erlangen Germany; ^3^ CCC WERA: Comprehensive Cancer Center Alliance WERA (CCC WERA) Erlangen Germany; ^4^ Bavarian Cancer Research Center (BZKF) Erlangen Germany; ^5^ Zentrum Personalisierte Medizin Erlangen (ZPM‐Erlangen) Erlangen Germany; ^6^ Institute of Human Genetics Universitätsklinikum Erlangen, Friedrich‐Alexander‐Universität Erlangen‐Nürnberg (FAU) Erlangen Germany; ^7^ Department of Nephropathology, Institute of Pathology Universitätsklinikum Erlangen, Friedrich‐Alexander‐Universität Erlangen‐Nürnberg (FAU) Erlangen Germany; ^8^ Department of Medicine 1 Universitätsklinikum Erlangen, Friedrich‐Alexander‐Universität Erlangen‐Nürnberg (FAU) Erlangen Germany; ^9^ Deutsches Zentrum Immuntherapie (DZI) Friedrich‐Alexander‐Universität Erlangen‐Nürnberg (FAU) Erlangen Germany; ^10^ Department of Radiation Oncology Universitätsklinikum Erlangen, Friedrich‐Alexander‐Universität Erlangen‐Nürnberg (FAU) Erlangen Germany; ^11^ Department of Surgery, Universitätsklinikum Erlangen Friedrich‐Alexander‐Universität Erlangen‐Nürnberg (FAU) Erlangen Germany; ^12^ Department of ENT, Head and Neck Surgery Universitätsklinikum Erlangen, Friedrich‐Alexander‐Universität Erlangen‐Nürnberg (FAU) Erlangen Germany; ^13^ Department of Obstetrics and Gynecology Universitätsklinikum Erlangen, Friedrich‐Alexander‐Universität Erlangen‐Nürnberg (FAU) Erlangen Germany; ^14^ Department of Urology and Pediatric Urology Universitätsklinikum Erlangen, Friedrich‐Alexander‐Universität Erlangen‐Nürnberg (FAU) Erlangen Germany; ^15^ Centre for Rare Diseases Erlangen Universitätsklinikum Erlangen, Friedrich‐Alexander‐Universität Erlangen‐Nürnberg (FAU) Erlangen Germany; ^16^ Department of Internal Medicine 5 Universitätsklinikum Erlangen, Friedrich‐Alexander‐Universität Erlangen‐Nürnberg (FAU) Erlangen Germany

**Keywords:** HRD, molecular Tumorboard, panCancer, TSO 500‐HRD

## Abstract

Alterations in Homologous Recombination Repair (HRR) Pathway genes have been found to be associated with HR‐Deficiency (HRD), which is an approved biomarker for PARP Inhibitor (PARPi) treatment. The aim of a Molecular Tumor Board (MTB) is to identify molecular alterations in cancer patients with advanced tumors that may suggest off‐label treatment options. So far, few studies have analyzed the presence of HRR gene mutations and their association with HRD outside of clinical studies. Currently, no data on HRD testing in the setting of a MTB have been published. For the present study, a cohort of 237 patients encompassing 24 different tumor entities was collected from the MTB of the Comprehensive Cancer Center Erlangen‐EMN. We show that an elevated Genomic Instability Score (GIS ≥42) can occur in samples with and without mutations in HRR‐related genes. Overall, 38.1% of cancer samples with *BRCA1/2* mutations, 10.9% of tumors with alterations in HRR genes other than *BRCA1/2*, and 4.3% of cancer samples without HRR gene mutations harbored an elevated GIS. Notably, our data show that various inactivating *BRCA1/2* mutations are not associated with an elevated GIS. Taken together, panCancer assessment of HRD in addition to *BRCA1/2* and other HRR gene mutational analysis is recommended to guide decisions regarding PARPi treatment. Further studies are needed to establish thresholds for GIS in non‐ovarian cancer entities. Finally, HRD can be observed in 4.3% of *BRCA1/2* and other HRR gene wildtype cancer samples, and may emerge as an independent biomarker for PARPi in the future.

## Graphical abstract

What's new?

Currently, no published data on homologous recombination deficiency (HRD) testing in the setting of molecular tumor boards exist. This study in a cohort of 237 patients encompassing 24 different tumor entities assessed by a molecular tumor board shows that inactivating alterations of *BRCA1*/*2* are not always associated with an elevated genomic instability score. Furthermore, HRD could be detected in various tumor entities harboring deleterious alterations in homologous recombination repair (HRR) genes other than *BRCA1*/*2*. HRD analysis should become part of comprehensive genomic profiling as it provides additional information for PARPi treatment recommendation in the setting of molecular tumor boards.

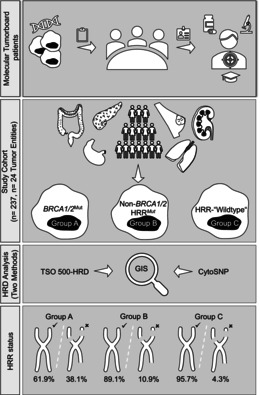

**Abbreviations**
ASCAT: Allele‐specific copy number analysis of tumors
BAF: B‐Allele frequency
CCC: Comprehensive Cancer Center
CCP: Comprehensive Cancer Panel
CGP: Comprehensive Genomic Profiling
CNA: Copy Number Alteration
dMMR: DNA mismatch repair deficient
FFPE: Formalin Fixed Paraffin Embedded
GIS: Genomic Instability Score
HRD: Homologous Recombination Deficiency
HRR: Homologous Recombination Repair
LOH: Loss Of Heterozygosity
LST: Large Scale Transition
MTB: Molecular Tumor Board
NMD: Nonsense mediated decay
PARP: Poly (ADP‐ribose)‐Polymerase
PARPi: PARP inhibitor
TAI: Telomeric Allelic Imbalance
TSO 500: TruSight Oncology 500 gene panel
TST170: TruSight Tumor 170 gene panel

## 1 INTRODUCTION

Mutational processes generate characteristic genomic signatures in cancer genomes, and comprehensive genomic profiling (CGP) of somatic mutations can be used to identify the predominant mutational processes in a given tumor tissue.^1^ This identification is increasingly relevant in clinical settings, since clinical response to drug treatments has been linked to the presence of mutational signatures, for example, response to immune checkpoint blockade in dMMR tumors^2^ and response to Poly (ADP‐ribose)‐Polymerase‐Inhibitors (PARPi) in Homologous Recombination deficient (HRD) tumors.^3^

On a technical level, HRD is determined by counting specific genomic rearrangements in a tumor associated with defects in the HRR pathway. Namely, genomic alterations that fulfill the criteria of either Loss Of Heterozygosity (LOH^4^), Telomeric Allelic Imbalances (TAI^5^) or Large Scale Transitions (LSTs^6^) are counted, and the sum of these alterations in a given tumor sample/genome is regarded as the Genomic Instability Score (GIS). The GIS is a continuous variable ranging from 0 to >100, whereas HRD is a qualitative parameter that has been defined as GIS ≥42 according to the distribution of GIS in cancer samples harboring a deleterious or suspected deleterious *BRCA1/2* mutation.^7^, ^8^, ^9^ So far, it is unknown whether the established threshold of GIS ≥42 for the determination of HRD in ovarian cancer is also applicable to other cancer entities. Moreover, other molecular alterations (e.g., *BRCA1* promoter hypermethylation) may cause HRD that cannot be detected by DNA sequencing of *BRCA1/2* and other HRR genes alone. PARPi are approved drugs in some cancer entities with *BRCA1/2* genomic alterations (ovarian cancer, breast cancer, pancreatic cancer and prostate cancer^10^, ^11^, ^12^, ^13^, ^14^) and also in ovarian cancer characterized by HRD.^15^ Early detection of *BRCA1/2* germline mutational status in breast cancer patients, for example, is crucial for identifying patients who could actually benefit from a PARPi therapy (summarized in^16^). Response to PARPi has initially been observed in ovarian cancer patients with alterations in the genes *BRCA1* and *BRCA2*,^17^, ^18^ but later studies reported that clinical response was also observed in patients with *BRCA1*/2 wildtype status.^19^, ^20^ A subset of these patients harbored alterations in other genes involved in the homologous recombination repair (HRR) pathway indicative of a possible HRD, and it has been proposed that the presence of HRD may be an effective biomarker to identify patients who respond to PARPi.

Molecular tumor boards (MTBs) have been increasingly established at tertiary cancer centers to employ CGP in patients with advanced cancer, to identify molecular targets for off‐label approaches or in a clinical study setting.^21^, ^22^ Regarding HRD and PARPi, patients with *BRCA1/2* altered cancers other than ovarian, breast, pancreatic, and prostate cancer may benefit from an off‐label use of PARPi.^23^, ^24^, ^25^ Furthermore, patients with HRR gene alterations other than *BRCA1/2* may also benefit from PARPi, but the effect of HRR gene alterations on the integrity of HRR is more difficult to predict compared to *BRCA1/2* gene alterations.^26^ Therefore, the present cohort was analyzed for the mutational status of *BRCA1*, *BRCA2*, and 20 further HRR genes which did already serve as inclusion criteria in a clinical study,^27^ were suggested in a review^28^ or are listed in the TSO 500‐HRD technical datasheet (Table 1).

**TABLE 1:** Overview of the selected 22 HRR associated genes.

Gene	Gene symbol (HGNC)	CCP	TST 170	TSO 500	Reference
Ataxia Telangiectasia Mutated	*ATM*	×	×	×	^26^, ^27^, ^a^
Ataxia Telangiectasia and Rad3‐Related	*ATR*		×	×	^27^, ^a^
Brca1‐Associated Ring Domain 1	*BARD1*		×	×	^26^, ^a^
Breast Cancer 1 Gene	*BRCA1*	×	×	×	^26^, ^27^, ^a^
Breast Cancer 2, Early Onset	*BRCA2*	×	×	×	^26^, ^27^, ^a^
Brca1‐Interacting Protein 1	*BRIP1*	×	×	×	^26^, ^27^, ^a^
Cyclin‐Dependent Kinase 12	*CDK12*	×	×	×	^26^, ^a^
Checkpoint Kinase 1	*CHEK1*		×	×	^26^, ^27^, ^a^
Checkpoint Kinase 2	*CHEK2*	×	×	×	^26^, ^27^, ^a^
Fa Complementation Group A	*FANCA*	×		×	^27^, ^a^
Fa Complementation Group C	*FANCC*			×	^27^, ^a^
Fa Complementation Group I	*FANCI*		×	×	^a^
Fa Complementation Group L	*FANCL*		×	×	^26^, ^a^
Nijmegen Breakage Syndrome 1	*NBN*		×	×	^27^, ^a^
Protein Phosphatase 2 Regulatory Subunit B Alpha	*PPP2R2A*		×	×	^26^
Partner and Localizer Of Brca2	*PALB2*	×	×	×	^26^, ^27^, ^a^
Rad50 Double‐Strand Break Repair Protein	*RAD50*			×	^a^
Reca, *E. coli*, Homolog Of	*RAD51*		×	×	^a^
Reca‐Like Protein	*RAD51B*		×	×	^26^, ^a^
Rad51 Homolog C	*RAD51C*		×	×	^26^, ^27^, ^a^
Rad51 Homolog D	*RAD51D*		×	×	^26^, ^27^, ^a^
DNA Repair and Recombination Protein Rad54‐Like	*RAD54L*		×	×	^26^, ^a^

*Note*: Of the 1427 cancer patients presented in the Molecular Tumor Board of the CCC Erlangen‐EMN between August 2016 and October 2023, 229 tumors harbored inactivating molecular alterations in at least one of the following genes: *ATM*, *ATR*, *BARD1*, *BRCA1, BRCA2, BRIP1*, *CDK12*, *CHEK1*, *CHEK2*, *FANCA*, *FANCC*, *FANCI*, *FANCL*, *NBN*, *PPP2R2A*, *PALB2*, *RAD50*, *RAD51*, *RAD51B*, *RAD51C*, *RAD51D* and *RAD54L*. Genes were selected according to a clinical study,^26^ a review^27^ and the Illumina TSO 500‐HRD technical datasheet (^a^, see last column of the table). The first column lists the full gene name, whereas in the second column the gene symbol according to HGNC is listed. The other three columns represent the gene content of the CCP‐, TST 170‐ or TSO 500 panel, respectively. A cross indicates that the respective gene panel contains the relevant HRR associated gene.

^a^ Illumina TSO 500‐HRD technical datasheet.

In the current study, we determined the GIS in association with the mutational status of *BRCA1*, *BRCA2*, and 20 other HRR‐related genes across 143 tumors from 24 cancer entities with HRR pathway alterations. Our aim was to evaluate whether HRR gene mutations are always associated with an increased GIS, which would support the rationale to suggest PARPi in an off‐label use setting when any HRR gene mutation is observed. Furthermore, we evaluated the GIS also in 94 cancer samples from 16 entities without any detectable possible inactivating molecular alteration of *BRCA1/2* or 20 other HRR genes as a control. Since no threshold for HRD has been established for most cancer entities, we aimed to compare GIS from tumors with HRR mutations to GIS from tumors without HRR mutations within the same entity for pancreatic‐, biliary tract‐, prostate‐, and colorectal cancer. Finally, all tumors with elevated GIS were screened for *BRCA1/2* promoter hypermethylation as an alternate mechanism for elevated GIS.

## 2 MATERIALS AND METHODS

### 2.1 Cohort

**FIGURE 1:**
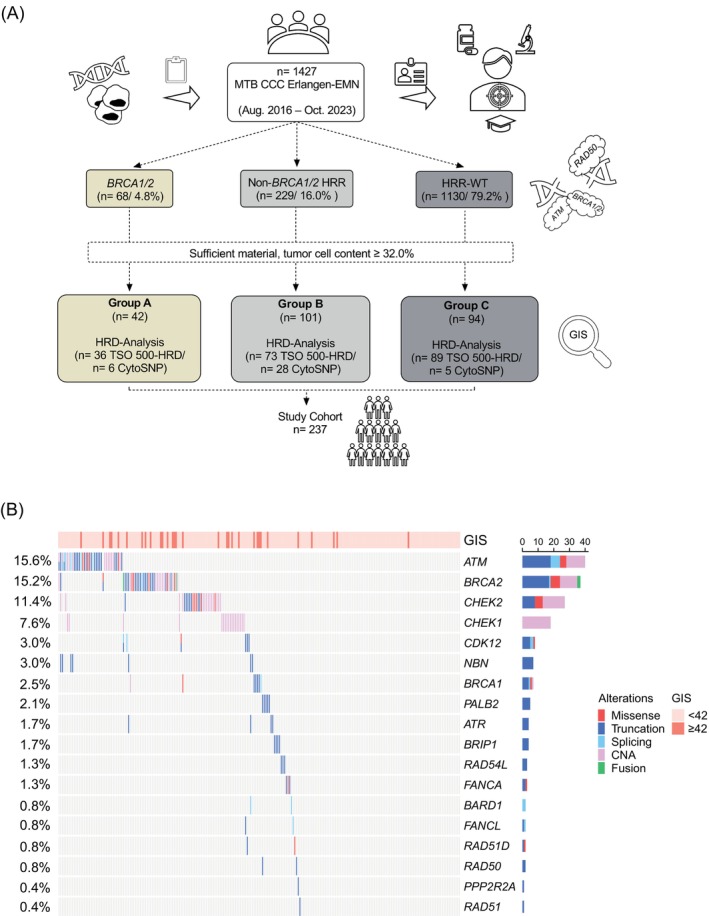
HRR‐gene alterations and GIS in the study cohort. (A) Workflow of the present study design. For the present study 1427 patients which had been presented in the Molecular Tumor Board of the CCC Erlangen‐EMN between August 2016 and October 2023 were analyzed for targetable molecular alterations using either Comprehensive Cancer Panel (CCP), TruSight Tumor 170 gene panel (TST 170) or TruSight Oncology 500 gene panel (TSO 500). The 1427 cases were screened for the presence of pathogenic or likely pathogenic inactivating molecular alterations affecting *BRCA1/2*. Of the 68 tumors, 42 eligible cases (sufficient amount of material, sufficient tumor cell content >32.0%) were analyzed for a possible HRD using either TSO 500‐HRD or the CytoSNP array (Group A, “Tumors with *BRCA1/2*”). Furthermore, the 1427 cases were screend for the presence of an inactivating molecular alteration affecting 20 HRR associated genes other than *BRCA1/2*. Of the 229 tumors, 101 eligible cases were analyzed for a possible HRD using either TSO 500‐HRD or the CytoSNP array (Group B, “Tumors with BRCA1/2 wildtype status and other HRR gene mutations”). Of the 1427 patients, 1130 patients did not harbor an inactivating molecular alteration in one of the 22 HRR‐related genes investigated. Of the 1130 tumors, 94 eligible cases were analyzed for a possible HRD using either TSO 500‐HRD or the CytoSNP array (Group C, “Tumors without *BRCA1/2* or other HRR gene mutations”). The total cohort which had been investigated in the present study comprised 237 patients. (B) Overview of the study cohort using OncoPrinter. Each column represents one patient. Rows indicate the respective molecular alteration. Samples are sorted in descending order according to their alteration frequency in 19 of the 22 HRR‐related genes investigated (see Section 2). Rows are sorted based on the frequency of the alterations in all samples and columns are reordered to visualize the mutual exclusivity between samples. Upper bar shows the Genomic Instability Score (light orange: GIS <42, orange: GIS ≥42) for each sample. Molecular alterations comprise missense (red), truncating (dark blue), splicing (light blue) and CNA (pink) on DNA‐level, as well as gene fusions, detected on RNA‐level (light green). Numbers on the left represent the alteration frequency of each gene in the present cohort. Numbers on the upper right indicate the total counts of each molecular alteration within the gene. Of the 237 patients, none of the cases harbored a molecular alteration in the genes *FANCC, FANCI, RAD51B and RAD51C*. Some of the graphical illustrations in (A) were used from Servier Medical Art licensed under CC BY 4.0 and adjusted accordingly (https://smart.servier.com/citation‐sharing/).

For the time period August 2016 to October 2023, a total of 1427 cancer patients underwent CGP with either Comprehensive Cancer Panel (*n* = 17, CCP, Qiagen, Hilden, Germany), TruSight Tumor 170 gene panel (*n* = 910, TST 170, Illumina, Inc., San Diego, CA) or TruSight Oncology 500 gene panel (*n* = 500, TSO 500, Illumina) in the context of the Molecular Tumor Board of the CCC Erlangen‐EMN (Figure 1A). Samples fulfilling necessary quality parameters (sufficient amount of material, sufficient tumor cell content >32.0%) were either analyzed in the setting of a research project using the TruSight Oncology 500 HRD assay (TSO 500‐HRD, Illumina, Inc., San Diego, CA, USA) or during routine diagnostics using the Infinium CytoSNP‐850 K v1.4 Bead Chips array (CytoSNP array, Illumina, Inc., San Diego, CA, USA). Although the TSO 500‐HRD and CytoSNP assay each differed in their performance rate regarding the determination of the GIS, both assays show a high concordance to the Myriad myChoice assay as validated in an interlaboratory comparison by a German consortium.^29^

From the 1427 cancer patients, 68 (4.8%) had tumors harboring inactivating molecular alterations in *BRCA1/2*. Of these 68 tumors, 42 eligible cases were analyzed for a possible HRD (Group A, “Tumors with *BRCA1/2* alterations”, either by TSO 500‐HRD, *n* = 36 or by CytoSNP, *n* = 6). Cases harboring an inactivating alteration in *BRCA1/2* were always assigned to group A, irrespective of an additional alteration in any of the other 20 HRR associated genes. From the 1427 cancer patients, 229 (16.0%) had tumors harboring inactivating molecular alterations in at least one of 20 other HRR related genes. HRR related genes other than *BRCA1* and *BRCA2* comprised the genes *ATM*, *ATR*, *BARD1*, *BRIP1*, *CDK12*, *CHEK1*, *CHEK2*, *FANCA*, *FANCC*, *FANCI*, *FANCL*, *NBN*, *PPP2R2A*, *PALB2*, *RAD50*, *RAD51*, *RAD51B*, *RAD51C*, *RAD51D* and *RAD54L* and were selected according to a clinical study,^27^ a review^28^ and the Illumina TSO 500‐HRD technical datasheet (Table 1). Of these 229 tumors, 101 eligible cases were analyzed for a possible HRD (Group B, “Tumors with *BRCA1/2* wildtype status and other HRR gene mutations”, either by TSO 500‐HRD, *n* = 73 or by CytoSNP, *n* = 28). Within the 1427 patients, 1130 (79.2%) patients did not harbor an inactivating molecular alteration in the 22 investigated HRR‐associated genes. Of these 1130 tumors, 94 eligible cases were analyzed for a possible HRD (Group C, “Tumors without *BRCA1/2* or other HRR gene mutations”, either by TSO 500‐HRD, *n* = 89 or by CytoSNP, *n* = 5). To enable a deeper cancer specific insight, Group C was enriched for cancer samples from pancreas, biliary tract, prostate and colorectum. The study cohort (*n* = 237) comprised 24 various tumor entities (Table 2). For 19 patients (8.0%), reports of germline screening in the local department of human genetics were available.

**TABLE 2:** Cohort characteristics.

Tumor type	GIS ≥42	GIS <42	Gene	SNV, *n* (%)	CNA, *n* (%)	Fusion, *n* (%)
Biliary duct (*n* = 35)	2	33	*ATM*	5 (14.3)	0.0	0.0
			*BRCA2*	3 (8.6)	1 (2.8)	0.0
			*CDK12*	1 (2.8)	0.0	0.0
			*CHEK2*	1 (2.8)	1 (2.8)	0.0
			*FANCL*	1 (2.8)	0.0	0.0
			*PALB2*	1 (2.8)	0.0	0.0
Bladder (*n* = 6)	1	5	*ATM*	0.0	1 (16.7)	0.0
			*BRCA2*	1 (16.7)	0.0	0.0
			*BRIP1*	1 (16.7)	0.0	0.0
			*CHEK1*	0.0	1 (16.7)	0.0
			*RAD51D*	1 (16.7)	0.0	0.0
Breast (*n* = 9)	4	5	*ATM*	0.0	2 (22.2)	0.0
			*BRCA1*	1 (11.1)	0.0	0.0
			*BRCA2*	0.0	1 (11.1)	0.0
			*CHEK1*	0.0	2 (22.2)	0.0
			*CHEK2*	0.0	1 (11.1)	0.0
			*NBN*	1 (11.1)	0.0	0.0
Cervix (*n* = 3)	0	3	*CHEK2*	1 (33.3)	1 (33.3)	0.0
			*CDK12*	1 (33.3)	0.0	0.0
			*RAD51D*	1 (33.3)	0.0	0.0
CNS (*n* = 3)	0	3	*BRCA2*	0.0	1 (33.3)	0.0
			*NBN*	1 (33.3)	0.0	0.0
Colorectal (*n* = 27)	1	26	*ATM*	2 (7.4)	0.0	0.0
			*ATR*	1 (3.7)	0.0	0.0
			*BRCA2*	2 (7.4)	0.0	0.0
			*BRIP1*	1 (3.7)	0.0	0.0
			*CHEK1*	0.0	2 (7.4)	0.0
			*CHEK2*	2 (7.4)	2 (7.4)	0.0
			*FANCA*	2 (7.4)	0.0	0.0
			*PALB2*	1 (3.7)	0.0	0.0
CUP (*n* = 14)	1	13	*ATM*	3 (21.4)	2 (14.3)	0.0
			*ATR*	1 (7.1)	0.0	0.0
			*BARD1*	1 (7.1)	0.0	0.0
			*BRCA1*	1 (7.1)	0.0	0.0
			*BRCA2*	1 (7.1)	1 (7.1)	0.0
			*CHEK2*	1 (7.1)	3 (21.4)	0.0
			*NBN*	1 (7.1)	0.0	0.0
			*RAD50*	1 (7.1)	0.0	0.0
Endometrium (*n* = 2)	1	1	*ATR*	1 (50.0)	0.0	0.0
			*BRCA2*	2 (100.0)	0.0	0.0
Esophagus (*n* = 4)	3	1	*ATM*	3 (75.0)	0.0	0.0
			*CHEK1*	0.0	1 (25.0)	0.0
Gallbladder (*n* = 2)	1	1	*ATM*	2 (100.0)	0.0	0.0
			*BRCA2*	1 (50.0)	0.0	0.0
Head and neck (*n* = 12)	1	11	*ATM*	1 (8.3)	1 (8.3)	0.0
			*BRCA2*	1 (8.3)	1 (8.3)	0.0
			*CHEK1*	0.0	2 (16.7)	0.0
			*CHEK2*	1 (8.3)	0.0	0.0
			*PALB2*	1 (8.3)	0.0	0.0
Kidney (*n* = 9)	1	8	*BRCA2*	3 (33.3)	0.0	0.0
			*BRIP1*	1 (11.1)	0.0	0.0
			*FANCA*	1 (11.1)	0.0	0.0
Liver (*n* = 1)	0	1	*ATM*	1 (100.0)	0.0	0.0
lung (*n* = 9)	2	7	*ATM*	0.0	1 (11.1)	0.0
			*BRCA1*	1 (11.1)	0.0	0.0
			*BRCA2*	0.0	1 (11.1)	0.0
			*CHEK1*	0.0	1 (11.1)	0.0
Ovary (*n* = 8)	4	4	*ATM*	0.0	1 (12.5)	0.0
			*BRCA1*	1 (12.5)	0.0	0.0
			*BRCA2*	1 (12.5)	0.0	0.0
			*CHEK2*	1 (12.5)	0.0	0.0
			*CDK12*	1 (12.5)	0.0	0.0
Pancreas (*n* = 25)	1	24	*ATM*	2 (8.0)	1 (4.0)	0.0
			*ATR*	1 (4.0)	0.0	0.0
			*BARD1*	1 (4.0)	0.0	0.0
			*BRCA2*	3 (12.0)	0.0	0.0
			*CHEK1*	0.0	2 (8.0)	0.0
			*CHEK2*	0.0	1 (4.0)	0.0
			*NBN*	2 (8.0)	0.0	0.0
			*PALB2*	1 (4.0)	0.0	0.0
			*RAD54L*	1 (4.0)	0.0	0.0
Prostate (*n* = 30)	4	26	*ATM*	5 (16.7)	1 (3.3)	0.0
			*BRCA2*	2 (6.7)	3 (10.0)	2 (6.7)
			*CDK12*	5 (16.7)	0.0	0.0
			*CHEK1*	0.0	2 (6.7)	0.0
			*CHEK2*	1 (3.3)	1 (3.3)	0.0
			*FANCL*	1 (3.3)	0.0	0.0
			*NBN*	2 (6.7)	0.0	0.0
			*PPP2R2A*	1 (3.3)	0.0	0.0
Salivary gland (*n* = 4)	0	4	*ATM*	2 (50.0)	1 (25.0)	0.0
			*BRCA2*	1 (25.0)	1 (25.0)	0.0
Skin, melanoma (*n* = 2)	0	2	*CHEK2*	1 (50.0)	0.0	0.0
Small intestine (*n* = 2)	0	2	*ATM*	1 (50.0)	0.0	0.0
			*CHEK2*	1 (50.0)	1 (50.0)	0.0
Soft tissue (*n* = 12)	1	11	*ATM*	3 (25.0)	1 (8.3)	0.0
			*BRCA1*	1 (8.3)	0.0	0.0
			*BRCA2*	0.0	1 (8.3)	0.0
			*CHEK1*	0.0	3 (25.0)	0.0
			*CHEK2*	2 (16.7)	1 (8.3)	0.0
			*RAD51*	1 (8.3)	0.0	0.0
Stomach (*n* = 6)	0	6	*BRCA2*	2 (33.3)	0.0	0.0
			*CHEK1*	0.0	1 (16.7)	0.0
			*PALB2*	1 (16.7)	0.0	0.0
			*RAD50*	1 (16.7)	0.0	0.0
			*RAD54L*	1 (16.7)	0.0	0.0
Testis (*n* = 2)	1	1	*CHEK1*	0.0	1 (50.0)	0.0
			*CHEK2*	0.0	1 (50.0)	0.0
Thyroid gland (*n* = 8)	1	7	*BRCA1*	1 (12.5)	0.0	0.0
			*BRIP1*	1 (12.5)	0.0	0.0
			*CHEK2*	1 (12.5)	1 (12.5)	0.0
Vulva (*n* = 2)	1	1	*BRCA1*	1 (50.0)	0.0	0.0
			*RAD54L*	1 (50.0)	0.0	0.0

*Note*: Distribution of GIS, tumor entity, mutated HRR gene and type of molecular alteration in 237 patients of the present study cohort.

Abbreviations: CNS, central nervous system; CUP, cancer of unknown primary.

### 2.2 Sample requirements and quality assessment

According to Illumina's internal limit of detection study for TSO 500‐HRD, a minimum tumor content of 32.0% needs to be present in the respective tumor sample to reach their claimed analytical specificity (Illumina technical datasheet TSO 500‐HRD). Further requirements for quantity specify a minimum of 40.0 ng DNA input material isolated from FFPE tissue as well as material within a predefined quality threshold. To check the prevalent DNA concentration, samples were quantified fluorometrically by using the dsDNA High‐Sensitivity Assay kit and Qubit 3.0 (Thermo Fisher Scientific, Waltham, USA). DNA quality was assessed by using the qPCR‐based Infinium FFPE QC kit of Illumina (San Diego, USA) and only DNA samples with a ΔCt ≤5 with regards to the Infinium FFPE kit's internal DNA control of good quality were used for further assessment.

### 2.3 Routine CGP using CCP‐, TST 170‐ or TSO 500‐ gene panel

Tumor material of all the 1427 patients had been analyzed using either CCP‐, TST 170‐, or the TSO 500 gene panel. The three used gene panels differ in their gene content (see Table 1). Therefore, 13 cases, originally analyzed by CCP‐ or TST170 gene panel, had to be reclassified using the TSO 500‐HRD panel (*n* = 11 cases of group C to group B, *n* = 2 of group B to group A, *n* = 1 of group C to group A). For sake of clarity, the definition of the study cohort refers to these final re‐classified cases. An H&E stain of formalin‐fixed, paraffin‐embedded cancer tissue was used for routine tumor diagnosis and for tumor cell content estimation. After microdissection of the tumor tissue, both DNA and RNA were isolated using standard techniques (Kit, Promega Maxwell). Generation of libraries, sequence alignments, and further bioinformatical analysis were performed according to the respective manufacturer's instructions and as already described elsewhere.^22^

### 2.4 Assessment of GIS using TruSight Oncology 500 HRD

For the assessment of GIS, 198 samples were analyzed within a retrospective research study using the TSO 500‐HRD panel in addition to the already applied routine CCP‐, TST170‐, or TSO 500 sequencing. For all samples, a total of 50.0 ng isolated FFPE DNA was used as starting material. First, the DNA was mechanically fragmented by using the Covaris ME220 instrument (Woburn, USA) following vendor recommendations to obtain average fragment sizes of 90–250 bp. Then, library Preparation was performed using Illumina's TSO 500 and TSO 500‐HRD add‐on kit following vendor's instructions. Final libraries were quantified by the dsDNA High‐Sensitivity Assay kit and Qubit 3.0 (Thermo Fisher Scientific) and normalized by Normalization Beads provided within the Illumina TSO 500 kit. Normalized libraries from eight tumor samples were pooled according to the manufacturer's instructions (NextSeq 550 Denature and Dilute Libraries Guide, Protocol G, Illumina) and sequenced in parallel on the NextSeq 550 instrument of Illumina using High Output kits v2.5 of 300 cycles. Average cluster densities of 200–220 K/mm^2^ were aimed for.

### 2.5 Data analysis of TSO 500‐HRD

Sequences obtained by NGS were subsequently aligned and analyzed using the docker‐based TSO 500 application (Illumina). Alterations were functionally annotated using the variant effect predictor (VEP, v. 104.3) by Ensembl^30^ and described using standard HGVS nomenclature. In addition, changes in gene copy numbers were determined by the CRAFT copy number variant caller (v1.0.0.12) algorithm incorporated in both the TSO 500‐ and TSO 500‐HRD application. The Genomic Instability Score (GIS) for each tumor sample was calculated according to the algorithm of Myriad Genetics by using the Illumina Basespace mounted DRAGEN TSO 500 Solid Eval App (Illumina). The sequencing coverage and quality statistics for each sample are summarized in Table S1.

### 2.6 Assessment of GIS using Infinium CytoSNP‐850K v1.4 BeadChips

For the assessment of GIS, 39 samples were analyzed during routine pathology diagnostics using the CytoSNP array in addition to the routine CCP‐, TST170‐, or TSO 500 sequencing. Up to 200.0 ng DNA was included in the analysis that was performed as recommended by Illumina. An initial quality control of DNA samples was performed by using Infinium FFPE QC kit of Illumina, and DNA samples with ΔCt ≤5.0 with regards to the Infinium FFPE kit's internal DNA control of good quality were processed further. After hybridization, the beadchips were scanned on the NextSeq 550 instrument of Illumina. Intensity data obtained by the NextSeq 550 were pre‐processed using GenomeStudio Software (V2.0, Illumina) to obtain logR and BAF values. A bioinformatics pipeline for logR and BAF values processing was developed in R v.4.2.2 and run in a dedicated conda environment on a remote server based on Ubuntu's 20.04.6 long‐term support (LTS) operating system. The workflow consisted of two steps: (1) allele‐specific copy number analysis of tumors by relying on ASCAT (v.3.1.1, segmentation penalty of =70, see^31^) and (2) determination of the levels of homologous recombination deficiency by relying on scarHRD v.0.1.1.^32^

### 2.7 Bisulfite pyrosequencing

All cases that harbored a GIS ≥30 and had no obvious molecular alteration in *BRCA1/2* as well as other HRR‐related genes were chosen for further bisulfite‐pyrosequencing. Additionally, we included 16 HRD‐positive samples with mutations in *BRCA1/2* as control. A total of 20.0 to 500.0 ng DNA was treated with sodium bisulfite using the EZ DNA Methylation‐Gold Kit (Zymo Research, USA). PCR amplification of the *BRCA1* and *BRCA2* target regions using already published primers was carried out applying standard PCR settings.^33^ Subsequently, pyrosequencing reactions were carried out using the PyroMark Gold Q24 Reagents (Qiagen) in a PyroMark Q24 Pyrosequencing System (Qiagen) according to the manufacturer's protocol (Pyrosequencing primers, BRCA1‐S 5′‐TTTGAGAGGTTGTTGTTTA‐3′ and BRCA2‐S 5′‐GGAGTAGTTGTGGTATTGTT‐3′). Quantification of CpG methylation was performed using the software PyroMark Q24 v.2.0.6 (Qiagen). The moderate amplification bias towards unmethylated alleles was corrected using the calibration data derived from a set of Epitect control DNA samples (Qiagen) and BiasCorrector software as previously described.^34^, ^35^

### 2.8 Data visualization and statistics

Figures and graphs were generated using GraphPad Prism version 9.5.1 for Windows (GraphPad Software, San Diego, California USA). Representation of the molecular profile of the study cohort was performed by relying on the ComplexHeatmap package (v. 2.13.1) within the R environment version 4.1.2.^36^ Graphical schemata were generated and adjusted accordingly using images from Servier Medical Art licensed under CC BY 4.0 (https://smart.servier.com/citation-sharing/) or pictograms using Powerpoint Version 2108 of Microsoft Office LTSC Professional Plus 2021. For statistical analysis, one‐way ANOVA, Fisher's exact test, or Mann–Whitney *U* test were applied. Values of *p* < 0.05 were considered statistically significant.

## 3 RESULTS

Genomic Instability Scores in three different molecular subgroups harboring inactivating alterations in either *BRCA1/2* (Group A), alterations in 20 other HRR associated genes (Group B) or no alteration in HRR associated genes (Group C), were assessed.

In total, the selected cohort comprised 237 patients (61.2% males, 38.8% females) with a mean age of 61.4 years encompassing 24 different tumor entities (Tables 2 and S2). Forty‐two cases could be assigned to group A, in which seven cases had co‐alterations in at least one of the other 20 HRR associated genes. One hundred one cases could be assigned to group B, in which 11 cases had co‐alterations in at least one of the other non‐*BRCA1/2* HRR associated genes. Ninety‐four cases could be assigned to group C harboring neither an inactivating alteration in *BRCA1/2* nor in any of the other 20 HRR associated genes. Overall, 31 of the 237 (13.1%) patients harbored a GIS ≥42. Of these, 16 cases (51.6%) could be assigned to group A, 11 cases (35.5%) could be assigned to group B, and four cases (12.9%) could be assigned to group C.

Inactivating pathogenic or likely pathogenic mutations were observed in 18 out of the possible 22 HRR genes investigated. Most of the cases harbored at least one inactivating molecular alteration in *ATM* (*n = 33*), *BRCA2* (*n = 33*), *CHEK2* (*n* = 24) or *CHEK1* (*n* = 17) (Figure 1B). Twenty‐four cases showed more than one mutation in one of the 22 HRR‐associated genes investigated. All types of alterations were detected in *ATM* and *BRCA2*, with the exception of fusions, which were detected only in *BRCA2*‐altered cases. Copy number alterations (CNAs) were the predominant alteration, mainly affecting *CHEK1* and *CHEK2* (18/51, 35.3% and 13/51, 25.5%).

### 3.1 Genomic instability score in tumors with *BRCA1/2* alterations (Group A)

**FIGURE 2:**
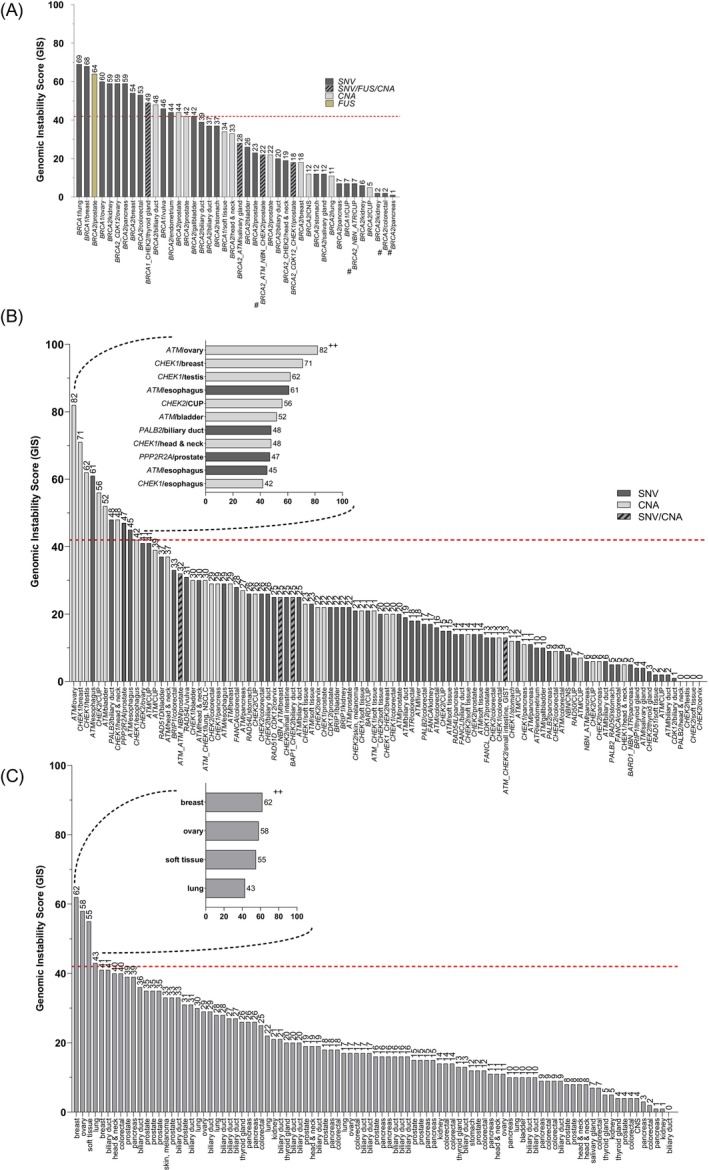
GIS across tumor entities in three different molecular subgroups. Molecular subgroups comprise (A) Tumors with *BRCA1/2* alterations (Group A), (B) Tumors with *BRCA1/2* wildtype status and other HRR gene mutations (Group B) and (C) Tumors without *BRCA1/2* or other HRR gene mutations (Group C, see Section 2). The y‐axis shows the Genomic Instability Score (GIS) of patients ranging from min. 0 to max. 82. On the x‐axis, the affected gene in the respective tumor entity is shown. Molecular alterations comprise SNVs (dark grey), CNAs (light grey), fusions (light brown) or a combination of SNVs + CNAs + Fusions (grey with black pattern). The red dashed line separates GIS positive (i.e., GIS ≥42, upper part) from GIS negative (i.e., GIS <42, lower part) cases. For (B): Three cases harboring a GIS of 0 were characterized by nonsense/missense mutations in the genes *CHEK2* and *PALB2*, whereas one case with a GIS of 0 was characterized by a *CHEK2* CN‐Loss. For (B, C): cases harboring a GIS ≥42 are highlighted in a separate graph in the respective upper left corner. ^#^Mismatch‐repair‐deficient, dMMR carcinomas, ^++^Hypermethylation of *BRCA1*.

Forty‐two cases with mutations in *BRCA1/2* had been selected, affecting 19 of 24 investigated tumor entities (Group A, Table 2 and Figure 2A). Of these, only 16 cases (38.1%) displayed an increased GIS (i.e., GIS ≥42, Figure 2A). On the other hand, 26 cases (61.9%) did not show an elevated GIS although harboring a pathogenic single nucleotide variant (SNV), CNA, or gene fusion in *BRCA1* or *BRCA2*. In detail, 35 patients harbored molecular alterations in *BRCA2*. Of these, six patients harbored co‐alterations in one of the 21 HRR genes. Seventeen cases harbored truncating mutations, six cases harbored missense mutations, and one case was affected by a splice site mutation. One patient harbored both a truncating alteration as well as a missense alteration. The remaining nine cases were characterized by *BRCA2* CN‐Loss. In addition, two cases showed a possible *BRCA2* inactivating gene fusion event at the RNA level (Figure 2A). Of the 35 *BRCA2* mutant tumors, only 11 tumors showed a GIS ≥42. These 11 tumors showed different mechanisms of a *BRCA2* gene inactivation (i.e., frameshift‐/missense mutations, CN alterations or a gene fusion). Truncating mutations in *BRCA2* were distributed all over the gene. Interestingly, six N‐terminally located frameshift mutations were not accompanied by an elevated GIS. In contrast to this, one far C‐terminally located truncating alteration was associated with an elevated GIS ≥42. Notably, four cancer samples with dMMR harbored a putative pathogenic mutation in *BRCA2* as a possible bystander effect not associated with an increased GIS (Figure 2A). One case with a gene fusion involving *BRCA2* was associated with a possibly defective function of the wildtype *BRCA2* protein possibly responsible for the observed GIS ≥42.

Of the 42 cases of group A, seven patients harbored molecular alterations in *BRCA1*. Of these, one case harbored a synchronous *CHEK2* CN‐Loss. Of the seven patients, five cases harbored truncating or splicing mutations, whereas one case harbored a missense loss of function mutation. In addition, one patient harbored a *BRCA1* CN‐Loss on the exon level (CNA: 0.7). Inactivating missense as well as nonsense mutations in both *BRCA1* and *BRCA2* seemed to be more frequently associated with an elevated GIS than CNAs, albeit reaching no statistically significance (*n* = 12/16 vs. *n* = 3/16, 75.0% vs. 18.8%, ns, *p* = 0.48, Fisher's exact test Figure 2A).

### 3.2 Genomic Instability Score in tumors with *BRCA1/2* wildtype status and other HRR gene mutations (Group B)

One hundred one cases with mutations in 20 HRR associated genes besides *BRCA1/2* had been selected, affecting all investigated tumor entities (Group B, Table 2 and Figure 2B). Of these, 11 cases (10.9%) had a GIS ≥42.

Taking a closer look, 35 patients harbored at least one inactivating alteration in the *ATM* gene comprising truncating/ splice‐ (*n* = 21) or missense‐ (*n* = 4) mutations, while *ATM* CN‐losses were observed in 11 patients. Of the *ATM* altered cases, four cases harbored a GIS ≥42. Twenty‐four cases harbored a molecular alteration in *CHEK2* comprising truncating/ splice‐ (*n* = 8), missense‐ (*n* = 5) or CN‐losses (*n* = 12). One case harboring a *CHEK2* CN‐Loss harbored a GIS ≥42. Seventeen patients harbored molecular alterations in *CHEK1*, strictly characterized by CN‐losses. Of the *CHEK1* altered cases, four cases harbored a GIS ≥42. Five patients each harbored inactivating, truncating mutations in *NBN* or *PALB2*. Of these, one *PALB2*‐mutant tumor harbored a GIS ≥42, whereas all other cases did show a GIS <42. Four patients each harbored either inactivating frameshift mutations in *BRIP1 or* truncating‐ (*n* = 4) or missense‐ (*n* = 1) mutations in *CDK12*. All of these tumors did show a GIS <42. Three patients each harbored either inactivating mutations in *FANCA* comprising truncating‐ (*n* = 2) and missense (*n* = 1) mutations, truncating mutations in *RAD54L*, or truncating mutations in *ATR*. All of these cases had a GIS <42. Two patients each harbored either inactivating splice‐site mutations in *BARD1*, inactivating mutations in *FANCL*, truncating mutations in *RAD50*, or inactivating mutations in *RAD51D* comprising truncating‐ (*n* = 1) and missense‐ (*n* = 1) mutations. All of these cases had a GIS <42. One patient each harbored either a truncating mutation in *PPP2R2A* or a truncating mutation in *RAD51*. The *PPP2R2A* mutant sample did show a positive GIS of 47, whereas the *RAD51* altered case harbored a GIS <42.

Of the 101 cases of group B, 11 cases harbored a synchronous co‐alteration in at least one other non‐*BRCA1/2* HRR associated gene (Figure 2B, grey bars with black lines). All of these 11 cases did show a GIS <42.

None of the patients harbored an inactivating molecular alteration in the genes *FANCC*, *FANCI*, *RAD51B*, and *RAD51C*.

Additional promotor hypermethylation analysis of the patient harboring an *ATM* CN‐loss characterized by the highest GIS of the present study (GIS: 82) did reveal a *BRCA1* DNA hypermethylation in the vicinity of the transcriptional start site. This observation is suggestive of a potential epigenetic driven mechanism of *BRCA1* gene inactivation and subsequent development of genomic instability (Figures 2B and S3). The other 10 cases with GIS ≥42 showed no *BRCA1/2* promoter hypermethylation, indicating that the respective HRR gene CNA or/and SNV may represent the main trigger for the elevated GIS.

### 3.3 Genomic Instability Score in tumors without *BRCA1/2* or other HRR gene mutations (Group C)

Forty‐nine cases, harboring no mutation in *BRCA1*/2 or any other of the selected 20 HRR genes, had been selected (Group C, Table 2 and Figure 2C). These samples were designated as “wildtype” control and were enriched for cancer samples of the pancreas (13 cases), biliary tract (22 cases), colorectum (12 cases) and prostate (15 cases). Notably, four out of 94 “wildtype” cases (4.3%) showed a positive GIS ≥42. All four cases belonged to different tumor entities.

Additional promoter hypermethylation analysis of these four cases revealed that a breast cancer sample harboring a GIS of 62 harbored a DNA promoter hypermethylation in the vicinity of the *BRCA1* transcriptional start site (Figures 2C and S3), while the remaining three cases displayed no hypermethylation.

### 3.4 Genomic Instability Scores across various tumor entities and histomorphologies

**FIGURE 3:**
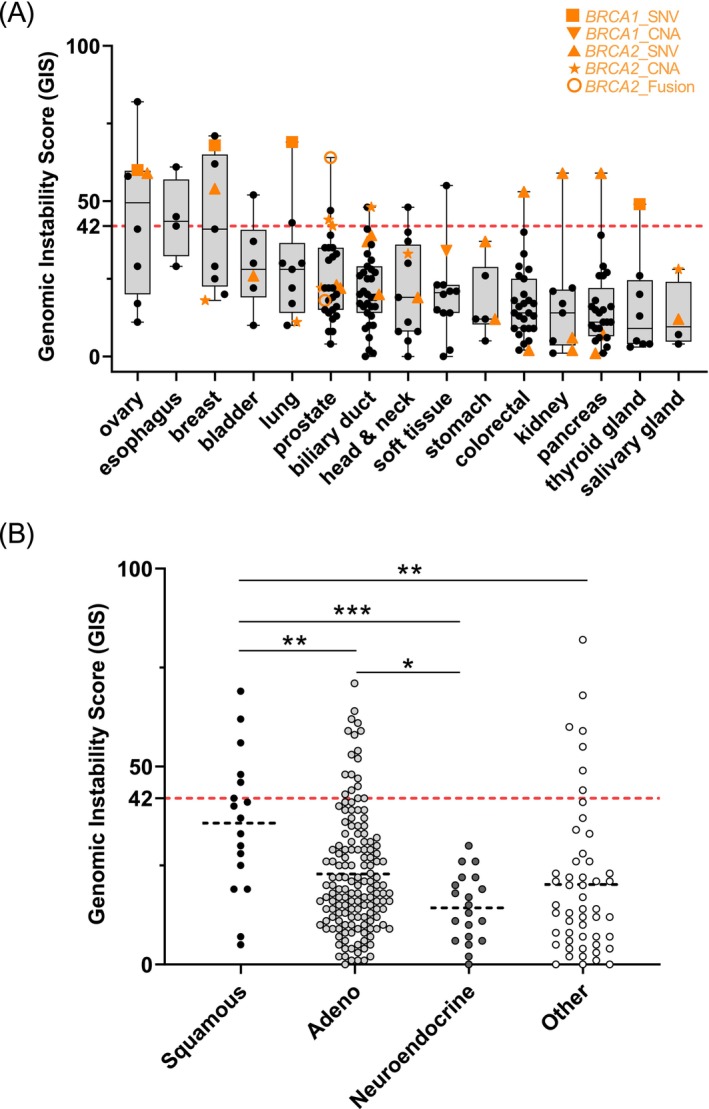
GIS across various tumor entities and histomorphologies. (A) The x‐axis shows various tumor entities of the present study represented by at least three patients. The y‐axis depicts the Genomic Instability Score (GIS) of patients ranging from minimum of 0 to maximum of 82. The dashed red line separates GIS negative (i.e., <42, lower part) from GIS positive cases (i.e., ≥42, upper part). Each black dot of the box plot represents a single case of the three molecular subgroups (Groups A–C, see Section 2). Orange color highlights tumor samples harboring *BRCA1/2* molecular alterations. *BRCA2* gene fusions are depicted as open circles (*BRCA2*_Fusion), *BRCA2* copy number losses are depicted as stars (*BRCA2*_CNA) whereas *BRCA1/2* nonsynonymous single nucleotide variants are depicted as squares (*BRCA1_SNV*) or triangles (*BRCA2*_SNV). One case harbored an exon‐specific CN‐loss in *BRCA1* (flipped orange triangle). (B) The y‐axis depicts the Genomic Instability Score (GIS) of patients ranging from minimum of 0 to maximum of 82. The dashed red line separates GIS negative (i.e., <42, lower part) from GIS positive cases (i.e., ≥42, upper part). On the x‐axis, four different histo‐morphology subtypes are depicted: Squamous carcinomas (black circles), Adenocarcinomas of various entities (light‐grey circles), hormone‐triggered neuroendocrine tumors (dark‐grey circles) as well as other morphological subtypes, for example, acinic carcinoma, adenoid‐cystic carcinoma, or leiomyosarcoma (open‐black circles). Statistical analysis was performed using one‐way ANOVA or Mann–Whitney *U* test.

Comparing the GIS values from the four tumor entities prostate‐, biliary tract‐, colorectal‐, and pancreatic cancer revealed that the median GIS was significantly different among these entities (prostate cancer: 22, range 4–64 versus biliary tract cancer: 20, 0–48 versus colorectal cancer: 14, 2–53 versus pancreatic cancer: 11, 1–59, *p* = 0.0241, One‐Way ANOVA, Figure 3A). Furthermore, breast‐ (median of 41, range 18–71), ovarian‐ (median of 49.5, 11–82) and esophageal‐ (median of 43.5, 29–61) cancer samples seemed to have higher GIS levels compared to the other cancer entities (median GIS of all other 21 entities: 18, range 0–69). Notably, out of four esophageal cancer samples, three samples had a GIS ≥42, although harboring no alterations in the genes *BRCA1/2*. Three of the four cases had inactivating alterations in *ATM* (*n = 2* GIS ≥42), whereas one case did harbor a *CHEK1* CN‐Loss (*n* = 1 GIS ≥42).

Irrespective of the site of origin, squamous cell carcinomas showed significantly higher GIS levels compared to adenocarcinomas (median 37 vs. 19, *p* = 0.0026 Figure 3B), neuroendocrine neoplasms (median 37 vs. 14, *p* = 0.0001) or other tumor entities (e.g., sarcomas, median 37 vs. 14, *p* = 0.0017, all Mann–Whitney *U* test). Furthermore, adenocarcinomas harbored significantly higher GIS levels compared to neuroendocrine neoplasms (median 19 vs. 14, *p* = 0.0203, Mann–Whitney *U* test). None of the patients in the present cohort diagnosed with a neuroendocrine tumor harbored an elevated GIS.

### 3.5 Association between germline status and Genomic Instability Score

**FIGURE 4:**
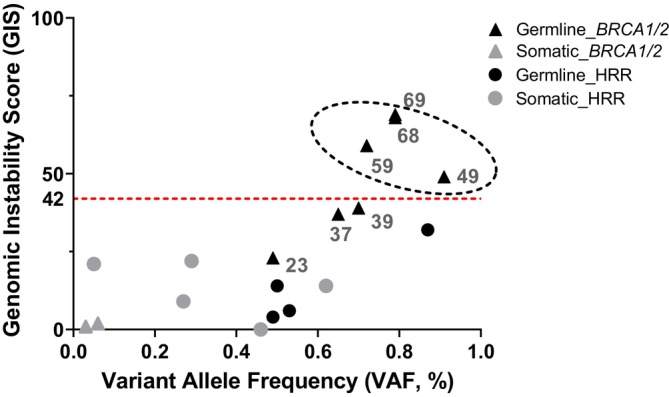
Correlation of GIS regarding germline vs. somatic mutation. The y‐axis depicts the Genomic Instability Score (GIS) of patients ranging from minimum 0 to maximum 82. The dashed red line separates GIS negative (i.e., <42, lower part) from GIS positive cases (i.e., ≥42, upper part). On the x‐axis, the variant allele frequency is shown in percentages ranging from 0.0 to 100.0%. Molecular alterations comprise solely SNVs of the affected genes. Triangles represent SNVs in the genes *BRCA1/2* either confirmed on germline‐ (black) or somatic‐level (grey). Circles reflect SNVs in HRR‐related genes either confirmed on germline‐ (black) or somatic‐level (grey). A black dotted circle highlights the GIS positive ≥42 germline mutated *BRCA1/2* samples. These cases comprised *n* = 3 *BRCA1* mutant cases and *n* = 1 *BRCA2* mutant case. Each of these *n* = 4 *BRCA1/2* mutations was present with a variant allele frequency of >50.0% in the respective tumor.

Of the 237 patients, 18 patients applied for germline testing in the local department of human genetics. Of these, seven patients could be assigned to group A harboring a confirmed germline mutation in *BRCA1* or *BRCA2*. Of the 18 patients, four cases could be assigned to group B harboring a confirmed germline mutation in either *ATM*, *BRIP1*, or *CHEK2*. Mutations harboring a high variant allele frequency of >50.0% were taken as read‐out for a possible loss‐of‐heterozygosity (LOH) of the second allele. Four of seven patients (57.1%) harboring a confirmed germline mutation in *BRCA1/2* and a possible LOH of the second allele were associated with a positive GIS ≥42 (Figure 4). Two cases with a confirmed germline mutation in *BRCA1/2* and a possible LOH of the second allele were not characterized by a positive GIS. Therefore, a germline alteration in *BRCA1/2* and a possible LOH of the second allele were not always associated with a GIS ≥42. Interestingly, none of the cases harboring a confirmed germline mutation in HRR‐related genes other than *BRCA1/2* was associated with a positive GIS (Figure 4).

## 4 DISCUSSION

In the current study, we analyzed a cohort of 237 patients for the presence of a possible HRD, characterized by a GIS ≥ 42. Tumors were separated into three groups, harboring either *BRCA1/2* alterations (Group A), HRR gene alterations other than *BRCA1/2* (Group B), and tumors without detectable HRR gene alterations (Group C).

Regarding group A, 38.1% of 42 tumors with *BRCA1/2* alterations displayed an elevated GIS, which is in line with previous studies.^37^ We showed that loss of function events in *BRCA1/2* comprising missense‐ and frameshift mutations, CN‐losses, as well as inactivating *BRCA2* gene fusions can be associated with an elevated GIS independent of the respective tumor entity. But on the other hand, 61.9% of tumors with possible *BRCA1/2* inactivating alterations were not associated with an elevated GIS ≥42. One explanation could be that far C‐terminally located truncating mutations in both *BRCA1/2* could escape nonsense‐mediated decay (NMD) of the affected mRNA and still lead to the expression of a functional BRCA1/2 protein. Furthermore, tumors harboring N‐terminally located truncating *BRCA2* mutations had a GIS < 42. Maybe these mutant proteins escape NMD and are truncated lacking all functional domains except the PALB2 binding domain.^38^ One could speculate that these truncated *BRCA2* proteins are still able to form the *PALB2*‐*BRCA1*‐*BRCA2* complex where both *PALB2* and *BRCA1* could compensate for the loss of *BRCA2* protein function. Furthermore, four cases harboring inactivating molecular alterations of *BRCA1/2* in the context of a microsatellite instable (MSI‐H) carcinoma were not associated with an elevated GIS and may thus represent a genomic bystander event. This is in line with a previous report which indicates that HRD and MSI‐high phenotypes are generally mutually exclusive events.^39^

Regarding group B comprising cases with inactivating alterations in HRR associated genes other than *BRCA1/2*, we observed a wide variety of alterations affecting all investigated tumor entities. Of the 24 tumor entities investigated, all entities harbored at least one alteration in one of the selected 22 HRR associated genes. An association of HRR gene alterations with GIS revealed that in 10.9% of tumor samples with HRR alterations, an elevated GIS was observed. Therefore, especially in this group and in the setting of a MTB, HRD testing may help to interpret the functional consequence and clinical relevance of the HRR gene alteration in individual cancer patients. Within group B, *ATM*, *CHEK2*, and *CHEK1* were the most frequently altered genes.

Most interestingly, four tumor samples of group C, representing 4.3% of that mixed cohort, showed a GIS ≥42. Detailed analysis of these four samples showed that even lower‐confident HRR genes were not altered (e.g., *ATRX*, *BAP1* or *MRE11* ^28^).

In addition, the present study addressed the question of whether *BRCA1* promotor hypermethylation can be associated with a GIS ≥42. We could show that a *BRCA1* promotor hypermethylation was identified in two tumor samples belonging to either group B or C. Together with the two samples with *BRCA2* fusion genes in group A, this observation demonstrates that genomic mutation analysis at the DNA level alone may not detect all tumors with HRD. Although the clinical relevance of HRD status as a predictor of PARPi therapy response has not been demonstrated in cancer entities other than ovarian cancer so far, it further emphasizes the benefit of additional HRD analysis compared to genomic mutation analysis alone to identify tumors with defects in the HRR pathway.

For the current study, we analyzed cancer samples from pancreatic‐, biliary tract‐, prostate‐ and colorectal cancer without HRR genomic alterations to get an estimate of the normal distribution of GIS in these samples. Although the small sample size for these cancer types in our study limits the generalizability of the findings, there seems to be a difference in the median GIS levels comparing these four entities. This is in line with a panCancer study employing an in‐silico approach on published data which demonstrated that prostate‐, biliary tract‐, colorectal‐ and pancreatic cancers did indeed show different median GIS levels and generally a lower GIS compared to ovarian cancer samples.^40^ Taken together, these results suggest that entity‐specific thresholds for HRD have to be established, and that a GIS ≥42 may not be a universal threshold for HRD in all cancer types. The present study design does not allow us to draw any conclusions on an entity‐specific GIS threshold as the sample size of most of the entities investigated is too low.The present study also investigated whether the respective histomorphology correlates with the calculated GIS. We showed that squamous cell carcinomas displayed higher GIS levels in general compared to adenocarcinomas or neuroendocrine neoplasms. This phenomenon has already been observed for lung adenocarcinomas showing a lower HRD score compared to squamous cell carcinomas of the lung.^40^

In the setting of a MTB, HRD detection reveals another important advantage. Concomitant quantification of GIS offers an alternate method to better interpret variants of unknown significance. Possibly inactivating molecular events like *BRCA2* gene fusions can be directly compared to the GIS level serving as a direct functional readout.

In summary, we showed the additional value of GIS analysis compared to HRR gene mutation analysis alone in the setting of a MTB. Our data show that not all tumors with HRR gene alterations will have a genomic scar phenotype and that mutational analysis alone will not identify all tumors with potential inactivation of the HRR system. Nevertheless, prospective clinical trials showing the benefit of HRD testing and treatment with PARPi in cancer entities other than ovarian cancer are needed.

Until clinical study data are available, we suggest that the additional information on GIS level better reflects the tumor biology in individual cancers and should be taken into consideration in the setting of a MTB when a recommendation for off‐label PARPi use is considered. Additionally, a thorough clinical follow‐up is needed to identify tumors that will benefit from PARPi in the setting of an MTB. Until certain gaps in the evidence, like entity‐specific GIS thresholds, are developed, it is not clear whether HRD has the potential to become a cancer‐agnostic biomarker for response to PARPi.

## AUTHOR CONTRIBUTIONS

**Christoph Schubart:** Conceptualization; data curation; formal analysis; investigation; methodology; software; supervision; validation; visualization; writing – original draft; writing – review and editing. **Lars Tögel:** Conceptualization; data curation; investigation; methodology; software; writing – review and editing. **Maria Giulia Carta:** Data curation; formal analysis; investigation; methodology; software; visualization; writing – review and editing. **Philip Hetzner:** Formal analysis; investigation; methodology; writing – review and editing. **Lina Helbig:** Formal analysis; investigation; methodology; writing – review and editing. **Charlotte Zaglas:** Formal analysis; investigation; methodology; writing – review and editing. **Maria Ziegler:** Formal analysis; investigation; methodology; writing – review and editing. **Robert Stöhr:** Funding acquisition; project administration; supervision; writing – review and editing. **Annett Hölsken:** Data curation; formal analysis; validation; visualization; writing – review and editing. **Juliane Hoyer:** Methodology; resources; writing – review and editing. **Fulvia Ferrazzi:** Methodology; software; visualization; writing – review and editing. **Clemens Neufert:** Investigation; resources; writing – review and editing. **Sebastian Lettmaier:** Investigation; resources; writing – review and editing. **Marianne Pavel:** Investigation; resources; writing – review and editing. **Henriette Golcher:** Investigation; resources; writing – review and editing. **Sarina K. Mueller:** Investigation; resources; writing – review and editing. **Florian Fuchs:** Investigation; resources; writing – review and editing. **Carla E. Schulmeyer:** Investigation; resources; writing – review and editing. **Matthias W. Beckmann:** Investigation; project administration; resources; writing – review and editing. **Bernd Wullich:** Investigation; resources; writing – review and editing. **Abbas Agaimy:** Investigation; resources; validation; writing – review and editing. **Andre Reis:** Investigation; resources; writing – review and editing. **Arndt Hartmann:** Project administration; resources; supervision; validation; writing – review and editing. **Norbert Meidenbauer:** Conceptualization; data curation; investigation; resources; validation; visualization; writing – review and editing. **Silvia Spoerl:** Conceptualization; formal analysis; investigation; resources; validation; visualization; writing – review and editing. **Florian Haller:** Conceptualization; funding acquisition; project administration; supervision; validation; visualization; writing – review and editing. **Evgeny A. Moskalev:** Conceptualization; data curation; formal analysis; investigation; methodology; software; validation; visualization; writing – review and editing.

## CONFLICT OF INTEREST STATEMENT

Arndt Hartmann received honoraria for lectures or consulting/advisory boards for Abbvie, Agilent, AstraZeneca, Biocartis, BMS, Boehringer Ingelheim, Cepheid, Diaceutics, Gilead, Illumina, Ipsen, Janssen, Lilly, Merck, MSD, Nanostring, Novartis, Pfizer, Qiagen, QUIP GmbH, Roche, Sanofi, 3DHistotech, and other research support from AstraZeneca, Biocartis, Cepheid, Gilead, Illumina, Janssen, Nanostring, Novartis, Owkin, Qiagen, QUIP GmbH, Roche, and Sanofi. Florian Haller received honoraria for lectures or consulting/advisory boards from AstraZeneca, BMS, Boehringer Ingelheim, Novartis, and research support from Illumina and Qiagen. Bernd Wullich received speaker honoraria from Janssen‐Cilag and MSD. Marianne Pavel received honoraria for presentations or consultancy/advisory boards from Novartis, AAA (Advanced Accelerator Applications), IPSEN, ITM, Serb, Boehringer Ingelheim, MSD, Sanofi, Recordati, Riemser, and Lilly. All other authors declare no conflict of interest.

## ETHICS STATEMENT

This study was approved by the Ethics Committee of the University Hospital of Erlangen with written informed consent from the patients (ethics committee statements 100_17 B from April 7, 2017, addendum from July 27, 2021).

## Supporting information


**FIGURE S3.** Supporting information.

**TABLE S1.** Supporting information.

**TABLE S2.** Supporting information.

## Data Availability

Data that support the findings of this study are available from the corresponding author upon request. Array data has been deposited at the European Genome‐phenome Archive (EGA), which is hosted by the EBI and the CRG, under accession number EGAD00010002736. Further information about EGA can be found on https://ega-archive.org. “The European Genome‐phenome Archive of human data consented for biomedical research” (https://doi.org/10.1093/nar/gkab1059).
