# Supplementary material for: Limited association between HRR gene alterations and HRD in molecular tumor board cancer samples: Who should be tested for HRD?
Source: Int J Cancer. 2025 Apr 25;157(5):964–79. doi: 10.1002/ijc.35457 (PMC12232594; doi:10.1002/ijc.35457)

Limited Association between HRR Gene Alterations and HRD in Molecular Tumor Board Cancer Samples: Who should be tested for HRD?

Christoph Schubart, Lars Tögel, Maria Giulia Carta, Philip Hetzner, Lina Helbig, Charlotte Zaglas, Maria Ziegler, Robert Stöhr, Annett Hölsken, Juliane Hoyer, Fulvia Ferrazzi, Clemens Neufert, Sebastian Lettmaier, Marianne Pavel, Henriette Golcher, Sarina K. Mueller, Florian Fuchs, Carla E. Schulmeyer, Matthias W. Beckmann, Bernd Wullich, Abbas Agaimy, Andre Reis, Arndt Hartmann, Norbert Meidenbauer, Silvia Spoerl, Florian Haller and Evgeny A. Moskalev

Table of Contents

1. Supplementary Table S1……………………………......(available in a separate file)

2. Supplementary Table S2……………………………(available in a separate file)

3. Supplementary Figure S3……………………………………………………....2

Supplementary Figure S3

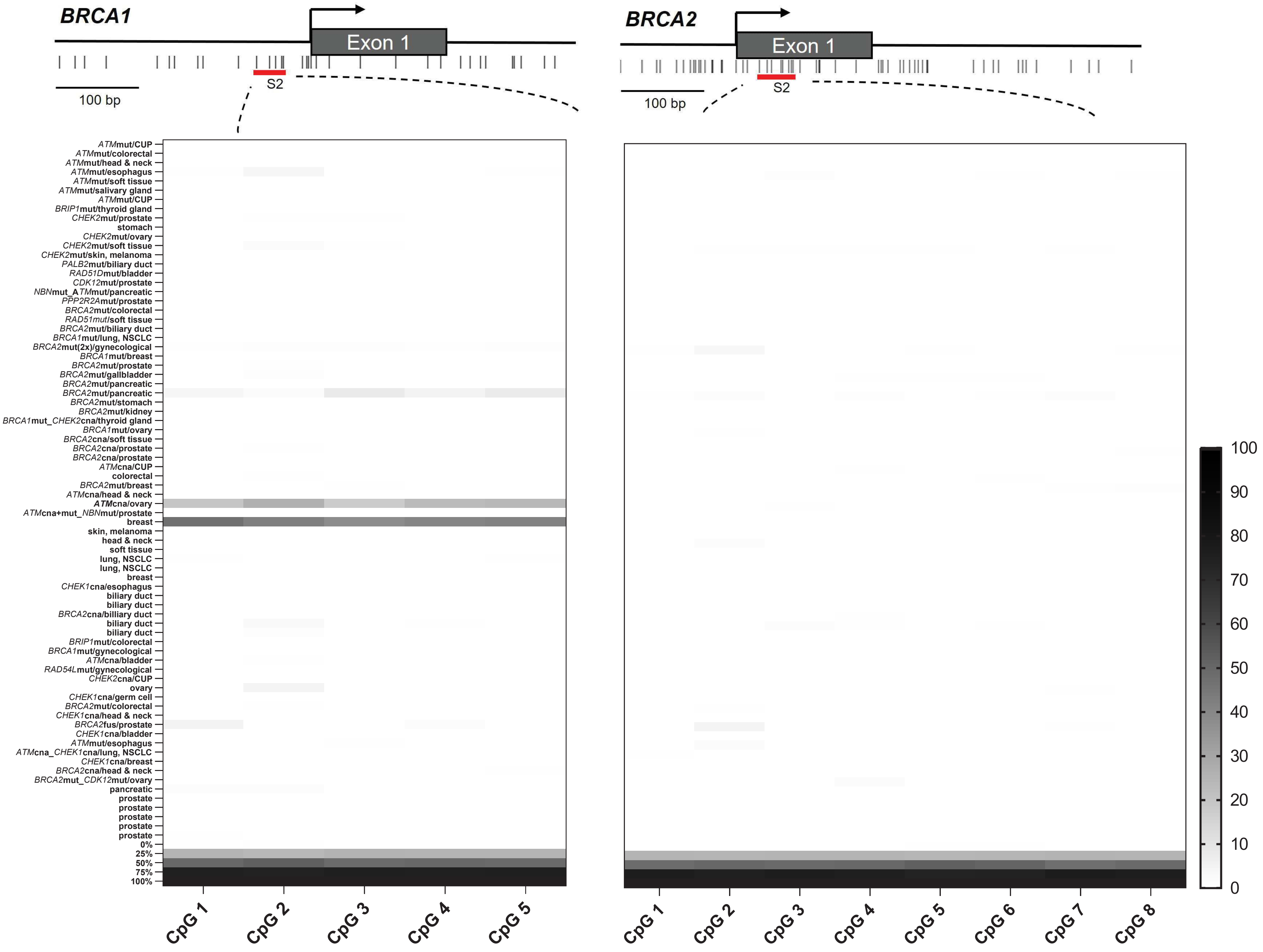

Supplement: Supplementary file 1 — FIGURE S3. Supporting information. [file IJC-157-964-s003.pdf]
